# Environmental Regulation, Environmental Decentralization, and Enterprise Environmental Protection Investment: Evidence From China

**DOI:** 10.3389/fpubh.2022.821488

**Published:** 2022-04-07

**Authors:** Li Liu, Guangqian Ren, Banghua He, Minna Zheng

**Affiliations:** ^1^School of Management, Henan University of Technology, Zhengzhou, China; ^2^Business School, Zhengzhou University, Zhengzhou, China; ^3^College of Agriculture and Rural Development, Renmin University of China, Beijing, China; ^4^School of Economics and Management, Hebei University of Technology, Tianjin, China

**Keywords:** environmental regulation, environmental decentralization, enterprise environmental protection investment, China, microeconomic behavior of enterprises

## Abstract

The microeconomic behavior of enterprises is influenced by the government system and its policies. In this article, we investigate how environmental regulation and environmental decentralization affect enterprise environmental protection investment using the data of China's listed companies from 2009 to 2020 and examine the effect of environmental decentralization on the relationship between environmental regulation and environmental protection investment. First, we find that there is a “U-shaped” relationship between environmental regulation and enterprise environmental protection investment. Second, the estimation results indicate that environmental decentralization can promote enterprise environmental protection investment. Finally, we further provide evidence to show that environmental decentralization has a negative moderating impact on the relationship between environmental regulation and enterprise environmental protection investment. Accordingly, the conclusion of this study is helpful to optimize the environmental decentralization management system, reasonably guide the local government behavior, alleviate the contradiction between environmental protection and economic development, and promote the green transformation of economic development mode.

## Introduction

China's economy has developed rapidly after the reforming and opening, but the extensive economic growth mode has also brought a series of problems, such as serious environmental pollution, low efficiency of resource utilization, ecosystem imbalance, and so on, which seriously endanger people's life and health (1). Data show that more than 80% of China's environmental pollutants are produced by enterprises (2), which have become the main producers of resource consumption and environmental pollution. As the main body of environmental pollution, enterprises have an unshrinkable responsibility for pollution control and should increase investment in environmental protection. With the continuous improvement of the strategic position of ecological environment protection, the concept of green development has become a common social consensus (3).

In the context of China's transition economy and ecological civilization construction, the economic losses and social contradictions caused by environmental pollution have become one of the main concerns of the Chinese government. Therefore, Chinese government has issued a number of environmental protection laws and regulations to strengthen the environmental responsibility of enterprises, which makes enterprises face more and more legal pressure. Notably, the 18th National Congress clearly proposes to strengthen the environmental protection and implement the environmental responsibility of local governments. Local governments not only formulate various economic policies, but also grasp a lot of resources (4), which have an impact on the decision-making of enterprises (5, 6). Enterprises with political relations can get more loans, more preferential tax rates, higher market share, and are easier to enter high barrier industries (7). In order to maintain the relationship with the government and get the support of the government, enterprises take practical actions to meet the expectations and requirements of the government (8). There is no doubt that environmental regulation (Reg), as an environmental management system, has an important impact on standardizing the environmental behavior of enterprises (9) and guides the investment decision-making direction of enterprises to a certain extent.

At the same time, China's economic decentralization (Dec) gives local governments financial autonomy, which makes local governments play an important role in ecological and environmental protection, resource conservation, and efficient utilization. Especially, after the 2008, the Chinese government has actively promoted the Dec of environmental power and targeted optimized the allocation of functions among governments at all the levels (10) to promote environmental and ecological protection and low-carbon production of enterprises (11). Therefore, environmental protection investment behavior of enterprises is also affected by environmental Dec.

In this regard, what is the impact of increasingly stringent environmental Reg on enterprises environmental protection investment? Does environmental Dec have an impact on enterprises environmental protection investment? Does the interaction between environmental Reg and environmental Dec affect the enterprises environmental protection investment? This article looks to answer these three key questions. The solution of these problems help to optimize the environmental Dec management system, reasonably guide the environmental protection behavior of local governments and enterprises, promote the ecological environment protection and efficient use of resources, alleviate the contradiction between environmental protection and economic development, and finally realize the green transformation of economic development mode.

The remainder of this article is the following. In Literature Review, we summarize the existing research. In Theoretical Background and Research Hypothesis, we introduce the theoretical background and discuss our hypotheses. In Empirical Design, we describe the empirical design, including data sources and sample selection, definition of variable, and model design. In Empirical Results, we present the descriptive statistics, correlation analysis, analysis of regression results, and robustness test. In Conclusion, we conclude by discussing conclusions.

## Literature Review

The literature on the relationship between environmental Reg, environmental Dec, and enterprise environmental protection investment (Epi) can be summarized from the following two aspects.

First, most scholars have not reached a consistent conclusion in the relevant research on the impact of environmental Reg on investment and development of enterprises. The main viewpoints can be summarized as “suppressionism” and “promotionism.” On one hand, “suppressionism” believes that environmental Reg increases the production cost of enterprises and inhibits the investment in environmental protection of enterprises. (12, 13). Herman (14) finds that environmental Reg inhibits the growth output of enterprises. Some studies have conducted that strict environmental control would not only increase production costs, but also reduce the competitiveness of enterprises (15). Thus, environmental Reg has a negative impact on productivity and hinders the overall development of enterprises (16, 17).

On the other hand, “promotionism” is based on the idea that reasonable environmental Reg can promote enterprises to increase the amount of investment in the field of environmental protection to a certain extent and expand the intensity of environmental protection investment (18, 19). The “Porter hypothesis” holds that the cost of production due to environmental control is offset by the search for alternative raw materials or the improvement of resource production efficiency (20, 21). The “Porter hypothesis” also believes that environmental Reg can bring about “innovation compensation effect,” which is conducive to the common improvement of environmental performance and economic performance (22, 23). The “Porter hypothesis” has also been verified by other scholars (24, 25). Ren et al. (26) believe that an effective environmental regulatory system is a key driving factor for improving regional ecological efficiency. Du and Li (27) find that environmental Reg restrains the extensive and intensive margins of export, where the negative impact on pollution intensive enterprises is greater than that on clean enterprises. Environmental Reg is an important means to solve economic development and environmental pollution, which plays an important role in green innovation (28) and high-quality development of China's economy (29, 30).

The second is the research on environmental Dec and its governance effect. Environmental Dec refers to the Dec of environmental governance rights and responsibilities from central government to local governments, which only implement environmental policies and regulations (31). With the increasingly prominent environmental problems in China, the research on environmental Dec and its governance effect is increasing (32). Until now, there has a long debate about the effect of environmental Dec. Most scholars analyze environmental Dec from the perspective of “bottom-to-bottom competition” and deem that the competition between local governments will lead to the “race to the bottom competition” of environmental Reg (33, 34). Scholars who support Dec believe that local governments can effectively provide public goods that meet the public expectations by virtue of information advantages compared with central governments (35). Therefore, environmental Dec is beneficial for local governments to formulate more stringent environmental Regs and expand the scale of environmental investment (36). In addition, some researchers have proposed that environmental Dec is not significantly reduce the strength of local environmental supervision and law enforcement (37–39).

However, some scholars believe that environmental Dec may also have some negative consequences. This is because environmental Dec may lead to inconsistent goals between local governments and the central government, which may lead to the phenomenon of “race to the bottom” (40). In order to increase fiscal revenue and get better political promotion opportunities, local officials pursue excessive capital investment. At this time, energy and pollution intensive industries are usually the preferred industries because the development of these industries can promote economic growth in a short period of time (41). However, this unsustainable economic growth model not only causes the deterioration of ecological environment and the waste of resources, but also has a serious negative impact, which further hinders the process of environmental protection investment (42). Therefore, there is still controversy about the impact of environmental Dec on local government's control of environmental pollution and improvement of environmental protection investment.

Different from previous research, this study has three contributions to the literature: First, this study examines the relationship between environmental Reg and enterprise Epi. Current research has not come to a consistent conclusion on the impact of environmental Reg on enterprise Epi. The research results of this article not only benefit the existing literature, but also have great policy significance for promoting the ecological environment protection and efficient use of resources in China.

Second, the relationship between environmental Dec and enterprise Epi is investigated in this study. Previous research is controversial about the impact of environmental Dec on local governments' investment in controlling environmental pollution and improvement of environmental protection investment. This article expands the current research on environmental Dec, which brings a new perspective for research on environmental protection investment.

Finally, this study incorporates environmental Reg, environmental Dec, and environmental protection investment in a theoretical and empirical research framework. Previous literature does not include environmental Reg, environmental Dec, and environmental protection investment in a theoretical and empirical research framework. The conclusions are helpful to understand the internal mechanism of environmental Reg, energy conservation, and emission reduction.

## Theoretical Background and Research Hypothesis

### Impact of Environmental Reg on Enterprise Epi

Enterprises' investment in environmental protection is a passive behavior and there is a general phenomenon of insufficient investment in environmental protection (43). This is because environmental protection investment not only has the characteristics of diversified objectives, long investment cycle, low return on investment and less economic benefits, but also occupies the investment of enterprises in other economic and productive projects. Obviously, enterprises usually do not take the initiative to invest in environmental protection without the government's environmental Reg policies. Environmental Reg is a rigid legal requirement for enterprises, which plays a role in guiding the environmental behavior of enterprises (44). The environmental protection department of the government has the right to punish the enterprises that pollute the environment and even force them to shut down (45). Enterprises adjust their environmental protection investment decisions according to government regulation policies (46). Therefore, the relationship between environmental Reg and enterprise Epi behavior is not a simple linear relationship, but a curve relationship, i.e., there is a threshold between environmental Reg and enterprise Epi.

Enterprises reduce the compliance rate of environmental standards and environmental protection expenditure when the intensity of environmental Reg is low. This is because the treatment of pollution emission involves the renewal of environmental protection facilities, the research and development of environmental protection technology, and the management of environmental protection organizations, which greatly increases the production cost of enterprises (47). At the same time, when the environmental control policy is relatively loose, the amount of environmental protection investment required by enterprises is often higher than the environmental taxes and fines when there is no or little investment (48). Even if the environmental Reg is strengthened to a certain extent, the enthusiasm of enterprises for environmental protection investment is not high and even prefer to pay relatively less environmental taxes and fines in order to reduce the amount of environmental protection investment (49, 50). At this time, the impact of environmental Reg on enterprise Epi is negative.

However, when environmental Reg is continuously strengthened and reaches a certain degree (threshold), the intensity of environmental Reg has reached a high level. The amount of environmental protection investment required by enterprises is not much different from environmental taxes and fines. Notably, with the improvement of the Chinese government's environmental control policies and the increasing efforts of environmental law enforcement, the environmental costs such as environmental taxes and fines to be paid for the emissions generated by the original system equipment have increased, which make the enterprises unbearable. In order to avoid being punished for failing to meet the environmental protection indicators, enterprises have to pay attention to environmental governance and environmental protection investment. At this time, there is a positive correlation between environmental Reg and enterprise Epi. Based on the above analysis, this article proposes the following hypothesis:

Hypothesis 1. There is a “U-shaped” relationship between environmental Reg and enterprise Epi.

### Impact of Environmental Dec on Enterprise Epi

Environmental Dec reflects the distribution of responsibilities and powers for environmental protection between the central and local governments of a country (51). The goal of the environmental protection department is to achieve effective environmental governance. The main functions include the implementation of environmental protection laws and regulations in the region, the preparation of environmental protection plans, the monitoring of environmental quality, and the investigation and handling of pollution accidents. With the improvement of environmental Dec, local governments have greater autonomy in environmental pollution control investment and more flexibility in environmental investment structure adjustment. In other words, environmental Dec strengthens the sense of responsibility of local governments for environmental protection. According to the local actual situation, local governments independently formulate environmental protection standards and design management systems to better manage local environmental affairs.

Specifically, environmental Dec may affect the decision-making of local governments in environmental governance and economic development (32). Furthermore, local governments guide enterprises to make environmental investment decisions by formulating scientific environmental standards (52). For example, local governments can directly affect the investment behavior and production behavior of enterprises by strengthening environmental supervision. In this way, local governments can further affect the environmental protection investment efficiency and energy consumption structure of enterprises (52). It can be seen from the above analysis that reasonable environmental Dec has fully stimulated the enthusiasm of local governments to provide regional environmental public services, especially in improving environmental quality and energy utilization. These favorable factors can effectively improve the environmental protection investment level of enterprises and reduce the sunk cost of governance. Based on the above analysis, this article proposes the following hypothesis:

Hypothesis 2. Environmental Dec has a positive impact on enterprise Epi.

### Moderating Effect of Environmental Dec

The relationship between environmental Reg and enterprise Epi is closely related to environmental Dec. The direction and structure of environmental Reg change with the change of local environmental affairs. In particular, with the continuous expansion of environmental management power, local governments relax the supervision of polluting enterprises in order to develop the regional economy or get promotion, which affect the environmental protection investment of enterprises. Therefore, it is of great significance to bring environmental Dec into the research on the relationship between environmental Reg and enterprise Epi.

On one hand, local governments actively reduce the environmental Reg standards in order to keep large polluting enterprises in the local area. Local governments often choose to sacrifice the ecological environment for short-term economic benefits in order to develop the regional economy or obtain promotion (53). Therefore, it is easy for local governments to choose to use their own rights to reduce the environmental Reg of large enterprises that cause serious environmental pollution, but bear most of the government's taxes. At this time, enterprises may intermittently stop the operation of environmental pollution control facilities, resulting in increasing pollution. This avoids the increase of production costs and the reduction of economic benefits of large enterprises due to environmental supervision.

On the other hand, rent-seeking behavior in the process of environmental Reg is inevitable. In order to reduce the negative impact of government environmental protection policies on production costs, enterprises (especially private enterprises) reduce the probability of being punished for pollution by establishing political relationship with local governments. At this time, the local government relaxes the requirements of the environmental supervision requirements for these enterprises and increases the rent-seeking space, which led to the pollution behavior of enterprises being covered up. Therefore, the greater the intensity of local environmental Dec, the more it inhibits the positive impact of environmental Reg on enterprise Epi. Based on the above analysis, this article proposes the following hypothesis:

Hypothesis 3. Environmental Dec has a negative moderating impact on the relationship between environmental Reg and enterprise Epi.

## Empirical Design

### Data Sources and Sample Selection

The initial sample comprises all of the Chinese listed companies during the period from 2009 to 2020. The reason for choosing 2009 as the first year is that China's Ministry of Ecological Environment and Shanghai Stock Exchange officially established the enterprise environmental information disclosure system in 2008. The final sample is obtained by screening this sample with the following conditions: (1) observations with abnormal data or with missing variables are removed; (2) ST and ^*^ST companies are removed; (3) financial, securities, and insurance companies are deleted; (4) companies listed in A-share market, B-share market, and H-share market at the same time are eliminated to avoid the consequences of differences in accounting standards, financing environment, and regulatory mechanism; and (5) companies with zero investment in environmental protection are excluded. Finally, 4,527 firm-year observations for 1,155 companies are obtained. Winsorize is applied to all the continuous variables at 1 and 99% quantiles to minimize the disturbance of abnormal observations.

The data of enterprises environmental protection investment are collected from the social responsibility report and sustainable development report publicly disclosed by listed companies on www.cninfo.com.cn. The data of environmental Reg and environmental Dec are obtained from the China Statistical Yearbook, the China Environmental Yearbook, and China Environmental Statistical Yearbook. The data of control variables are derived from China Stock Market, Wind Database, and the China Stock Market and Accounting Research (CSMAR) Database.

### Definition of Variable

#### Dependent Variable

Enterprise environmental protection investment: Enterprise Epi is the dependent variable that this article focuses on. The definition of enterprise Epi has not been unified in academic. Most scholars agree that enterprises carry out special economic activities while considering environmental and social benefits to prevent pollution and protect the environment (43). This study uses the ratio of total environmental protection investment to total assets to measure the scale of environmental protection investment, which effectively reduces the impact of different enterprise scale on it (54, 55).

#### Independent Variable

Environmental Reg: Environmental Reg reflects a country's demand for green environmental protection. The government formulates corresponding policies to regulate market economic activities and coordinates economic growth and environmental protection by protecting ecological environment and preventing industrial pollution (56). According to previous research, this article measures environmental Reg by the ratio of the total investment in regional industrial pollution control to the gross industrial product of each region. The higher the value means that the greater the degree of environmental Reg.

Environmental Dec: There are two main reasons why it is difficult to measure environmental Dec. First, it is difficult to find a suitable indicator to measure the degree of environmental Dec due to the differences of environmental Reg systems in different countries (57). Second, the definition of environmental Dec system in reality is very complex. The complex relationship between government departments has brought great difficulties to the measurement of environmental Dec (58). Third, in addition to environmental policies, regional environmental quality may also be affected by tax policies, energy policies, industrial policies, and other related policies (59).

According to the existing literature, most scholars judge whether a country is decentralized or centralized from the perspective of legal system (58, 60). However, these indicators do not reveal the nature of environmental Dec. Based on this consideration, this article refers the research of recent studies by Peng (61) and Wu et al. (34). We use the dynamic changes of employees in the central and local environmental protection systems to measure the level of environmental Dec. As for the number of environmental employees not disclosed in some years, we use the data of the last year to deduce. The specific formula is as follows:


(1) $$\begin{array}{c} ed_{it}=\left[\frac{lep_{it}/lpop_{it}}{nep_{it}/npop_{t}}\right]\times [1-(gdp_{it}/gdp_{t})] \end{array}$$

where *i* and *t* denote region and time, respectively; *ed*_*it*_ is the level of environmental Dec in the year *t* of region *i*; *nep*_*it*_ represents the total number of environmental protection systems in the country; *lep*_*it*_ represents the total number of environmental protection systems in each country; *npop*_*it*_ is the total population of the country; and *lpop*_*it*_ is the total population of each country. In addition, considering that there may be more employees in the environmental protection system in economically developed areas, the degree of environmental Dec may be greatly affected by the level of regional economic development. In this regard, [1−(*gdp*_*it*_/*gdp*_*t*_)] is added as the scale factor of economic development level to reduce the endogenous problems, in which*gdp*_*it*_ represents the gross domestic product (GDP) of each region and*gdp*_*t*_ represents the GDP of *t* year. The larger the index means that the larger the relative scale of local environmental protection agency, which the greater the degree of local government participation in environmental affairs, the higher the degree of environmental Dec. On the contrary, it means that the degree of environmental Dec is lower.

#### Control Variable

Enterprise Epi is affected by many factors, some explanatory variables must be controlled to avoid the error caused by those variables. Following the literature, we introduce the explanatory variables of enterprise characteristics, financial level and internal governance structure as control variables. Enterprise characteristics include three variables: enterprise scale, enterprise nature, and listing age. Financial level includes four variables: operating cash flow, operating performance, cash holding level, and financial leverage. The internal governance structure includes four variables: ownership concentration, management shareholding ratio, board size, and independent director proportion. In addition, annual dummy variables are also used as control variable. The description of all the variables is shown in Table 1.

**Table 1:** Definition of variables.

**Category**	**Name**	**Code**	**Definition**
Dependent variable	Enterprise environmental protection investment	*Epi*	Total environmental protection investment / total assets
Independent variable	Environmental regulation	*Reg*	Total investment in regional industrial pollution control / the gross industrial product of each region
	Environmental decentralization	*Dec*	$\left[\frac{lep_{it}/lpop_{it}}{nep_{it}/npop_{t}}\right]\times [1-(gdp_{it}/gdp_{t})]$
Control variable	Enterprise scale	*Size*	The natural logarithm of the total assets at the end of the year
	Enterprise nature	*Nature*	The value is 1 for state-owned enterprises and 0 for private enterprises
	Listing age	*Age*	The number of years the company has been listed
	Operating performance	*Roa*	Net profit / total assets
	Cash holding level	*Cash*	Total monetary capital at the end of the year / total assets at the end of the year
	Financial leverage	*Lev*	Year end total liabilities / year end total assets
	Operating cash flow	*Oper*	Net operating cash flow / total assets
	Management shareholding ratio	*M-share*	Number of shares held by management / total shares
	Board size	*Bod*	Natural logarithm of the number of directors
	Independent director proportion	*Ind*	Number of independent directors / number of directors
	Ownership concentration	*First*	Shareholding ratio of the largest shareholder
	Year	*Year*	Virtual variable

### Model Design

In order to investigate the relationship between environmental Reg and enterprise Epi, this article has constructed empirical models in Formula (2). If β_2_ in Formula (2) is positive and significant, it means that hypothesis 1 is verified.


(2) $$\begin{array}{l} \begin{array}{c} Epi_{it}=\beta_{0}+\beta_{1}Re\;g_{it-1}+\beta_{2}Re\;g^{2}{}_{it-1}+\sum^{\text{​}}Control_{it}+ \end{array}\\ \;\begin{array}{c} \sum^{\text{​}}Year\_Dummy+\varepsilon \end{array} \end{array}$$

In order to investigate the relationship between environmental Dec and enterprise Epi, this article has constructed empirical models in Formula (3). If β_1_ in Formula (3) is positive and significant, it means that hypothesis 2 is verified.


(3) $$\begin{array}{l} \begin{array}{c} Epi_{it}=\beta_{0}+\beta_{1}Dec_{it-1}+\sum^{\text{​}}Control_{it}+ \end{array}\\ \;\begin{array}{c} \sum^{\text{​}}Year\_Dummy+\varepsilon \end{array} \end{array}$$

In order to investigate the joint impact of environmental Dec and environmental Reg on enterprise Epi, this article has constructed empirical models in Formula (4) and Formula (5).


$$\begin{array}{l} Epi_{it}=\beta_{0}+\beta_{1}Re\;g_{it-1}\\ +\beta_{2}Re\;g^{2}{}_{it-1}+\beta_{3}Dec_{it-1}+\beta_{4}Re\;g_{it-1}\times Dec_{it-1}\\ \;=+\sum \\ Control_{it}+\sum Year\_Dummy+\varepsilon \end{array}$$

$$\begin{array}{l} Epi_{it}=\beta_{0}+\beta_{1}Re\;g_{it-1}+\beta_{2}\\ Re\;g^{2}{}_{it-1}+\beta_{3}Dec_{it-1}+\beta_{4}Re\;g_{it-1}\times Dec_{it-1}\\ \;=+\beta_{5}\\ Re\;g^{2}{}_{it-1}\times Dec_{it-1}+\sum Control_{it}\\ +\sum Year\_Dummy+\varepsilon \end{array}$$

Considering the lag of environmental Reg and environmental Dec on the results, we use the environmental Reg and environmental Dec with a lag period to regress with enterprise Epi.

## Empirical Results

### Descriptive Statistics

As a summary, the descriptive statistics of variables are given in Table 2. As we can see, the average enterprise *Epi* is 0.0044, the maximum value is 0.0332, and the minimum value is 0. The difference between the maximum value and the minimum value is about 1,613 times, which indicates that there are large individual differences in the sample. The average value is greater than the median, which indicates that the scale of environmental protection investment of most enterprises in the sample has reached the average level. The median of environmental *Reg* and environmental *Dec* is less than the average, which indicates that most of the sample enterprises are facing a low level of environmental Reg intensity and environmental Dec. The mean value of enterprise nature (*Nature*) is 0.2136, which indicates that about 21% of the samples are from state-owned enterprises and private enterprises account for a large proportion. The sample distribution of operating performance (*Roa*), cash holding level (*Cash*), financial leverage (*Lev*), and operating cash flow (*Oper*) shows that the average value of the financial level of the sample enterprises is close to the median, indicating that these variables are nearly normal distribution and the data have a certain representativeness. The analysis of m share shows that most of the enterprises in the sample have higher management share than the average level and equity incentive plays a certain role. The mean and median of the proportion of independent directors (*Ind*) are more than one-third, which meet the requirements of listed companies for the proportion of independent directors in board members.

**Table 2:** Description statistics of variables.

**Variable**	**Minimum**	**Maximum**	**Mean**	**Median**	**Standard deviation**
*Epi*	0.0000	0.0332	0.0044	0.0021	0.0058
*Reg*	0.0007	0.0155	0.0031	0.0024	0.0024
*Dec*	0.5331	2.1609	0.7782	0.7517	0.2266
*Size*	20.8901	27.0181	23.4877	23.4406	1.2712
*Nature*	0.0000	1.0000	0.2152	0.0000	0.4081
*Age*	3.0000	27.0000	16.9000	17.0000	6.4172
*Roa*	−0.1356	0.2255	0.0394	0.0303	0.0527
*Cash*	0.0199	0.5853	0.1548	0.1295	0.1080
*Lev*	0.1199	0.8786	0.5034	0.5273	0.1729
*Oper*	−0.1198	0.2654	0.0639	0.0600	0.0664
*M-share*	0.0000	0.5342	0.0407	0.0000	0.1118
*Bod*	6.0000	17.0000	9.4626	9.0000	2.0973
*Ind*	0.3333	0.6435	0.3726	0.3333	0.0598
*First*	11.4325	82.5162	40.9791	40.7535	16.2017

Then, following the research of Hosseini and Rezvani (62), this article uses the Kaiser–Meyer–Olkin (KMO) to make a preliminary analysis of the samples before the empirical analysis. The KMO index is 0.581, which shows that the sample size obtained in this article is sufficient.

### Correlation Analysis

Table 3 provides the results of main variables' Pearson correlation coefficients. As shown in Table 3, environmental *Reg* is positively correlated with enterprise *Epi* at the level of 1% and environmental *Dec* is positively correlated with enterprise *Epi* at the level of 10%. This means that the previous hypothesis has been preliminarily verified. At the same time, the absolute value of correlation coefficient between each variable is <0.5, which indicates that there is no serious multicollinearity in the model. Thus, the variable selection in this article is reasonable. It was further tested by the variance inflation factor (VIF) value.

**Table 3:** Correlation matrix of variables.

**Variable**	***Epi***	***Reg***	***Dec***	***Size***	***Nature***	***Age***	***Roa***	***Cash***
*Epi*	1							
*Reg*	−0.198***	1						
*Dec*	0.135*	0.321***	1					
*Size*	0.097	−0.006	0.083	1				
*Nature*	0.023	0.159***	0.322***	0.063	1			
*Age*	−0.113**	0.067	−0.002	0.079	0.037	1		
*Roa*	0.041*	−0.232***	−0.048	−0.022	−0.069	−0.114**	1	
*Cash*	−0.196***	−0.145***	−0.084	−0.097*	−0.015	0.043	0.204**	1
	*Lev*	*Oper*	*M-share*	*Bod*	*Ind*	*First*		
*Lev*	1							
*Oper*	−0.220***	1						
*M-share*	−0.198***	0.058	1					
*Bod*	0.116**	−0.027	−0.117**	1				
*Ind*	0.013	0.047	−0.117**	−0.310***	1			
*First*	0.073	0.198***	−0.275***	−0.034	0.104*	1		

***, **, and * indicate significant at the level of 1, 5, and 10% respectively.

### Analysis of Regression Results

According to the results of the Hausman test, we utilize panel fixed effect method to investigate the relationship between environmental Reg, environmental Dec, and enterprise Epi. The regression results of each model are shown in Table 4.

**Table 4:** Regression result.

**Dependent variable**	**(1)**	**(2)**	**(3)**	**(4)**
	***Epi***	***Epi***	***Epi***	***Epi***
*Reg*	−0.5094** (−2.1425)		−0.1820 (−0.2256)	−0.1279 (−0.1668)
*Reg* ^2^	60.2289*** (3.4553)		71.4688*** (2.7267)	53.3635* (1.9386)
*Dec*		0.0081** (2.2256)	0.0137*** (3.0125)	0.0122*** (2.6695)
*Dec × Reg*			−0.9426 (−1.1935)	−0.3929 (−0.4875)
*Dec × Reg^2^*				−29.2597** (−2.2745)
*Size*	0.0002 (0.2645)	0.0009 (0.7625)	0.0014 (1.2536)	0.0013 (1.1268)
*Nature*	0.0008 (0.3458)	0.0016 (0.4737)	0.0007 (0.2153)	0.0002 (0.0128)
*Age*	−0.0023** (−2.0246)	−0.0016* (−1.8615)	−0.0021* (−1.8156)	−0.0020* (−1.7489)
*Roa*	0.0085* (1.7337)	0.0049* (1.7858)	0.0011* (1.8225)	0.0019* (1.7397)
*Cash*	−0.0066* (−1.9312)	−0.0108** (−2.0567)	−0.0104** (−2.1176)	−0.0122** (−2.3825)
*Lev*	−0.0039* (−1.7325)	−0.0054* (−1.7067)	−0.0046* (−1.7285)	−0.0059* (−1.7335)
*Oper*	0.0008 (0.1933)	0.0069 (1.1959)	0.0052 (0.9058)	0.0063 (1.1128)
*M-share*	0.0199* (1.7925)	0.0192* (1.8180)	0.0211* (1.8259)	0.0215* (1.8668)
*Bod*	0.0001 (0.5986)	0.0002 (0.0125)	0.0001 (0.0665)	0.0002 (0.0879)
*Ind*	0.0150** (2.2697)	0.0116* (1.7134)	0.0090 (1.5616)	0.0105* (1.8288)
*First*	0.0006* (1.7231)	0.0007* (1.7496)	0.0006 (1.3937)	0.0008* (1.7467)
*Year*	Control	Control	Control	Control
*_cons*	0.0308 (0.9734)	0.0013 (0.0502)	0.0062 (0.1522)	0.0035 (0.0826)
*F*	6.2264***	4.2135***	4.2086***	4.3513***
*R^2^*	0.1403	0.1337	0.1859	0.2092

***, **, and * indicate significant at the level of 1, 5, and 10% respectively.

### Results of Environmental Reg on Enterprise Epi

We investigate the relationship between environmental Reg and enterprise Epi and the results are shown in column (1). As can be seen, the regression coefficient of environmental *Reg* is −0.5094, which is significantly negative at the level of 5%. The quadratic regression coefficient of environmental *Reg*^2^ is 60.2289, which is significantly positive at the level of 1%. Therefore, there is a “U-shaped” relationship between environmental Reg and enterprise Epi. In other words, before the degree of environmental Reg reaches its turning point, the improvement of environmental Reg inhibits the improvement of enterprise Epi. This conclusion verifies Hypothesis 1. The reason that environmental Reg is actually a kind of cost burden of enterprises. Enterprises driven by economic interests accept mild environmental punishment to pursue short-term interests when enterprises are faced with light environmental tax burden or environmental supervision. These enterprises convert environmental protection investment funds into production funds to obtain short-term benefits and make up for the losses caused by environmental punishment.

Furthermore, the inflection point of “U-shaped” curve can be calculated and the inflection point value is 0.0042. The median of environmental Reg faced by the sample enterprises is 0.0021, which is less than the inflection point. This finding shows that the vast majority of enterprises are located on the left side of the “U-shaped” curve. That is to say, the environmental Reg intensity faced by most enterprises in China has not yet reached the inflection point level and the environmental Reg intensity ratio faced by listed companies is relatively low. As environmental Reg has a negative impact on the scale of enterprise Epi, increasing the intensity of environmental Reg to the right of the inflection point will help to increase enterprise Epi. When the level of environmental Reg is on the right side of the inflection point, enterprises are afraid of environmental protection costs such as huge environmental taxes and severe environmental penalties. They have to increase Epi and carry out technological innovation to reduce environmental pollution and realize the legitimacy of enterprise production. At this time, environmental Reg and Epi are positively correlated.

### Results of Environmental Dec on Enterprise Epi

The results of column (2) in Table 4 reflect the impact of environmental Dec on enterprise Epi. As can be seen, the regression coefficient of environmental *Dec* is 0.0081, which is significant at the level of 5%. Thus, there is a positive correlation between environmental Dec and enterprise Epi. The higher the environmental Dec is, the more favorable it is for enterprises to integrate the concept of environmental protection into investment activities, which Hypothesis 2 has been verified. The results clearly show that the improvement of environmental Dec will enhance the Epi of enterprises. When the right of environmental governance is delegated to the local government, the local government has the information advantage of closely monitoring the Epi of enterprises. According to the actual situation of different regions, local governments in different regions formulate their own environmental protection policies, the frequency of environmental monitoring, and the intensity of punishment for violations. Only in this way, enterprises can be more targeted to promote investment in environmental protection and effectively solve the problem of environmental pollution in the region.

### Results of the Moderating Effect of Environmental Dec

The results of column (3) and column (4) in Table 4 show the impact of environmental Dec on the relationship between environmental Reg and enterprise Epi. As can be seen, the coefficient of interaction between environmental Reg and Dec (*Dec* × *Reg*) is −0.9426; however, the negative impact is not significant. The coefficient of interaction between secondary term of environmental Reg and the environmental Dec (*Dec* × *Reg*^2^) is −29.2597, which is significant at the 5% level. The results indicate that environmental Dec has a negative moderating impact on the relationship between environmental Reg and enterprise Epi, which verifies Hypothesis 3. Meanwhile, the results imply that the impact of environmental Dec on the relationship between environmental Reg and Epi changes with the intensity of environmental Reg.

The inhibition of environmental Dec on environmental Reg can be explained by rent-seeking theory. Environmental Dec makes the local government bear the responsibility of protecting the environment. However, local governments can be affected by the promotion and performance appraisal. In order to obtain political achievements, local governments provide “umbrella” to some large profit and tax enterprises that produce high environmental pollution. They even reduced the environmental supervision of these enterprises, resulting in these enterprises not paying attention to Epi. In addition, senior managers of enterprises are easy to form interest alliance with local officials when they seek political connection from local governments. Local officials can get benefits including money and promotion and enterprises will not be punished for reducing Epi under the protection of local government. At this time, environmental Dec plays a negative moderating role in the relationship between environmental Reg and enterprise Epi.

### Robustness Test

In order to ensure the robustness of the above regression results, we test the robustness in the following ways.

First, in order to alleviate the problems caused by the different measurement methods of the dependent variable, following research of Tang et al. (43) and Yang et al. (3), this article uses “enterprise's new Epi scale/average total assets” to measure the enterprise's *Epi*2. Table 5 column (1)–(4) shows the regression result. The results mean that the regression results are consistent with the above results after changing the measurement method of dependent variable, which shows that the regression results are robust.

**Table 5:** Robustness test.

**Dependent variable**	**(1)**	**(2)**	**(3)**	**(4)**	**(5)**	**(6)**	**(7)**	**(8)**
	***Epi*** **2**	***Epi*** **2**	***Epi*** **2**	***Epi*** **2**	***Epi***	***Epi***	***Epi***	***Epi***
*Reg*	−0.5839** (−2.0758)		−0.1626 (−0.2389)	−0.2314 (−0.3415)	−0.5285** (−2.0126)		−0.1967 (−0.3561)	−0.2528 (−0.2637)
*Reg* ^2^	58.8418*** (2.7537)		77.8003*** (2.8785)	44.2574 (1.5524)	57.3679*** (3.2540)		68.2689*** (2.6925)	58.3978* (1.9256)
*Dec*		0.0047* (1.7758)	0.0097** (2.1354)	0.0080* (1.7877)		0.0079** (2.1258)	0.0210*** (3.0235)	0.0285*** (2.6597)
*Dec × Reg*			−0.8477 (−1.1456)	−0.2531 (−0.3496)			−0.7589 (−1.1681)	−0.3268 (−0.5365)
*Dec × Reg^2^*				−42.4413*** (−3.1668)				−27.3689** (−2.2034)
*Size*	0.0007 (0.7283)	0.0012 (1.0668)	0.0008 (0.7025)	0.0010 (0.9157)	0.0008 (0.2387)	0.0010 (0.5869)	0.0011 (1.3256)	0.0009 (1.1039)
*Nature*	0.0023 (1.1186)	0.0032 (1.2285)	0.0017 (0.6238)	0.0037 (1.3714)	0.0045 (0.4027)	0.0022 (0.4569)	0.0007 (0.2023)	0.0007 (0.0212)
*Age*	−0.0030*** (−2.6925)	−0.0028** (−2.1297)	−0.0035*** (−2.6445)	−0.0031** (−2.4088)	−0.0018** (−2.0125)	−0.0028* (−1.8345)	−0.0028* (−1.8367)	−0.0031* (−1.7526)
*Roa*	0.0105* (1.7576)	0.0005* (1.7589)	0.0051* (1.8485)	0.0011* (1.8236)	0.0046* (1.7228)	0.0058* (1.7275)	0.0025* (1.8034)	0.0022* (1.7278)
*Cash*	−0.0082** (−2.1368)	−0.0084* (−1.8181)	−0.0089* (−1.9525)	−0.0102** (−2.2853)	−0.0027* (−1.8463)	−0.0142** (−2.4468)	−0.0197** (−2.1239)	−0.0106** (−2.3120)
*Lev*	−0.0030* (−1.8969)	−0.0054* (−1.7678)	−0.0042* (−1.8618)	−0.0049* (−1.7637)	−0.0025* (−1.7023)	−0.0039* (−1.7152)	−0.0034* (−1.7891)	−0.0055* (−1.7526)
*Oper*	0.0056 (1.2697)	0.0045 (0.7886)	0.0057 (1.0145)	0.0042 (0.7559)	0.0026 (0.3785)	0.0064 (1.1253)	0.0068 (0.9289)	0.0057 (1.1264)
*M-share*	0.0136* (1.8125)	0.0076* (1.8485)	0.0110* (1.7465)	0.0115* (1.7846)	0.0129* (1.7385)	0.0126* (1.8387)	0.03058* (1.8369)	0.0226* (1.8338)
*Bod*	0.0003 (1.1934)	0.0002 (0.7869)	0.0001 (0.4971)	0.0002 (0.7718)	0.0001 (0.5028)	0.0002 (0.0564)	0.0003 (0.0726)	0.0002 (0.0759)
*Ind*	0.0075* (1.8946)	0.0111* (1.8456)	0.0075* (1.7118)	0.0102* (1.7859)	0.0107** (2.2269)	0.0125* (1.7687)	0.0078 (1.5726)	0.0126* (1.8311)
*First*	0.0009** (2.4666)	0.0001** (2.3865)	0.0009** (2.1128)	0.0001** (2.4258)	0.0006* (1.7015)	0.0007* (1.7564)	0.0006* (1.7368)	0.0008* (1.7526)
*Year*	Control	Control	Control	Control	Control	Control	Control	Control
*_cons*	0.0658* (1.7452)	0.0711* (1.7035)	0.0748* (1.7829)	0.0720* (1.7578)	0.0412 (0.9635)	0.0017 (0.0642)	0.0056 (0.1759)	0.0029 (0.0877)
*F*	5.1267***	3.6485***	3.5886***	3.8479***	3.2453***	3.6725***	3.4567***	3.9785***
*R^2^*	0.1754	0.1813	0.2067	0.2448	0.1689	0.2016	0.2113	0.1868

***, **, and * indicate significant at the level of 1, 5, and 10% respectively.

Second, the regression results may be affected by the difference of sample time. Therefore, we replace the study sample. The data of independent variable and control variables are selected from 2010 to 2017, while the data of dependent variables are selected from 2009 to 2020 for robustness test. The regression results are shown in Table 5 column (5)–(8). It can be seen that there is no change in the regression results, indicating that the research conclusion of this study is relatively reliable.

## Conclusion

According to empirical results in this article, three main conclusions can be drawn:

(1) It is found that environmental Reg is an important influencing factor of enterprise Epi. Therefore, Chinese governments at all the levels need to clarify their responsibilities for environmental protection. The government should strengthens environmental supervision and standardizes the economic behavior of enterprises by improving the environmental protection legal system and environmental protection regulations.

(2) By exploring the relationship between the environmental Dec and the enterprise Epi, this article suggests that the environmental Dec significantly affects the enterprise Epi. Therefore, the environmental governance power of local government in environmental management needs to be further strengthened. In particular, local governments should be given more freedom in environmental management system, personnel structure, and environmental governance investment, which can give full play to the information advantages of local governments in environmental management.

(3) Environmental Dec has a negative moderating impact on the relationship between environmental Reg and enterprise Epi. This means that the central government should strengthen the supervision of local governments on environmental pollution control and incorporate environmental control into the performance appraisal system of local officials, which can stimulate their enthusiasm for environmental protection. It is necessary to further improve the environmental supervision system and environmental performance evaluation, which can strengthen environmental protection monitoring.

## Data Availability Statement

Publicly available datasets were analyzed in this study. This data can be found here: https://www.wind.com.cn/.

## Author Contributions

LL and GR designed this research and the model, analyzed the data, wrote the article, and provided editorial supports. BH and MZ obtained inference and analyzed the data. All the authors cooperated to revise the article. All authors contributed to the article and approved the submitted version.

## Funding

This study is supported by High Level Talent Fund Project of Henan University of Technology (2021SBS32), Philosophy and Social Science Planning Project in Henan Province of China (Project No. 2020BJJ056), Excellent Youth Scientific Research Team Cultivation Plan of Humanities and Social Sciences of Zhengzhou University (2020-QNTD-01), General Project of Humanities and Social Science Research in Colleges and Universities in Henan Province (2021-ZZJH-366), and Innovation Team Support Program of Henan University of Technology (32410000).

## Conflict of Interest

The authors declare that the research was conducted in the absence of any commercial or financial relationships that could be construed as a potential conflict of interest.

## Publisher's Note

All claims expressed in this article are solely those of the authors and do not necessarily represent those of their affiliated organizations, or those of the publisher, the editors and the reviewers. Any product that may be evaluated in this article, or claim that may be made by its manufacturer, is not guaranteed or endorsed by the publisher.

## References

[1] Akbar A Jiang X Qureshi MA Akbar M. Does corporate environmental investment impede financial performance of Chinese enterprises? The moderating role of financial constraints. Environ Sci Pollut Res. (2021) 2021:58007–17. 10.1007/s11356-021-14736-2 34101121

[2] Shen HB Xie Y Chen ZR. Environmental Protection, Corporate Social Responsibility and Its Maket Response-Case Study Based on the Environment Pollution Incident of Zijin Mining Group Co. China Ind Econ. (2012) 141–151. (in Chinese). 10.19581/j.cnki.ciejournal.2012.01.014

[3] Yang L Qin H Gan Q. Internal control quality, enterprise environmental protection investment and finance performance: an empirical study of china's A-Share heavy pollution industry. Int J Environ Res Public Health. (2020) 17:6082. 10.3390/ijerph17176082 32825596 PMC7503461

[4] Zhou LA. Governing China' s local officials: an analysis of promotion tournament Model. Econ Res J. (2007) 7:36–50.

[5] Chen S Sun Z Tang S Wu D. Government intervention and investment efficiency: evidence from China. J Corp Financ. (2011) 17:259–71. 10.1016/j.jcorpfin.2010.08.004

[6] Gan C Zou J Wang J. Term of local officials, enterprise resource acquisition and excess capacity. China Ind Econ. (2015) 3:44–56. (in Chinese). 10.19581/j.cnki.ciejournal.2015.03.004

[7] Faccio M. Politically connected firms. Am Econ Rev. (2006) 96:369–86. 10.1257/000282806776157704

[8] Song Z Nahm AY Zhang Z. Partial state ownership, political connection, and financing: evidence from Chinese publicly listed private sector enterprises. Emerg Mark Financ Trade. (2017) 53:611–28. 10.1080/1540496X.2015.1097920

[9] Sun T Feng Q. Evolutionary game of environmental investment under national environmental regulation in China. Environ Sci Pollut Res Int. (2021) 28:53432–43. 10.1007/s11356-021-14548-4 34031832

[10] Millimet DL. Environmental federalism: a survey of the empirical literature. IZA Discuss Papers. (2013) 27:1930–8. 10.2139/ssrn.2372540

[11] Li C Chandio AA He G. Dual performance of environmental regulation on economic and environmental development: evidence from China. Environ Sci Pollut Res. (2021) 29:3116–30. 10.1007/s11356-021-15466-1 34389948

[12] Hancevic PI. Environmental regulation and productivity: the case of electricity generation under the CAAA-1990. Energy Econ. (2016) 60:131–43. 10.1016/j.eneco.2016.09.022

[13] Chang KC Wang D Lu YY Chang W Ren GQ Liu L. Environmental Regulation, Promotion Pressure of Officials, and Enterprise Environmental Protection Investment. Front Public Health. (2021) 9:724351. 10.3389/fpubh.2021.724351 34422755 PMC8377723

[14] Herman B. Why has productivity slowed down. Data Resour Can Bus Res. (1981) 8:10–4.15242160

[15] Wang S Sun X Song M. Environmental regulation, resource misallocation, and ecological efficiency. Emerg Mark Financ Trade. (2021) 57:410–29. 10.1080/1540496X.2018.152956031673967

[16] Sun H Liu Z Chen Y. Foreign direct investment and manufacturing pollution emissions: a perspective from heterogeneous environmental regulation. Sustain Dev. (2020) 28:1376–87. 10.1002/sd.2091

[17] Ai H Hu Y Li K. Impacts of environmental regulation on firm productivity: evidence from China's Top 1000 Energy-Consuming Enterprises Program. Appl Econ. (2021) 53:830–44. 10.1080/00036846.2020.1815642

[18] Wen HW Deng WF Guo QN. Assessing the effects of the Environmental Protection Tax Law on heavily polluting firms in China. PLoS ONE. (2021) 16:0261342. 10.1371/journal.pone.0261342 34914798 PMC8675716

[19] Chen CX Lin RF Zheng X Zheng XQ Li YY. Environmental Regulations and Corporate Green Investment: Evidence From Heavy Polluting Companies in China. E3S Web Conf. (2021) 275:02051. 10.1051/e3sconf/202127502051

[20] Jin W Zhang H Liu S Zhang H. Technological innovation, environmental regulation, and green total factor efficiency of industrial water resources. J Clean Prod. (2019) 211:61–9. 10.1016/j.jclepro.2018.11.172

[21] Zhang P Wu F Guo YL Ma HF. Does enforcement matter in promoting corporate environmental investment: Evidence from Chinese private firms. J Clean Prod. (2022) 337:130432. 10.1016/j.jclepro.2022.130432

[22] Porter ME. America's Green Strategy. Sci Am. (1991) 264:193–246. 10.1038/scientificamerican0491-168

[23] Porter ME Linde C. Toward a new conception of the environment-competitiveness relationship. J Econ Perspect. (1995) 9:97–118. 10.1257/jep.9.4.97

[24] Manello A. Productivity growth, environmental regulation and win-win opportunities: The case of chemical industry in Italy and Germany. Eur J Oper Res. (2017) 262:733–43. 10.1016/j.ejor.2017.03.058

[25] Ramanathan R He Q Black A Ghobadian A Gallear D. Environmental regulations, innovation and firm performance: a revisit of the Porter hypothesis. J Clean Prod. (2017) 155:79–92. 10.1016/j.jclepro.2016.08.116

[26] Ren S Li X Yuan B Li D Chen X. The effects of three types of environmental regulation on eco-efficiency: a cross-region analysis in China. J Clean Prod. (2018) 173:245–55. 10.1016/j.jclepro.2016.08.113

[27] Du W Li M. Influence of environmental regulation on promoting the low-carbon transformation of China's foreign trade: Based on the dual margin of export enterprise. J Clean Prod. (2019) 244:118687. 10.1016/j.jclepro.2019.118687

[28] Wang F Feng LL Li J Wang L. Environmental Regulation, Tenure Length of Officials, and Green Innovation of Enterprises. Int J Environ Res Public Health. (2020) 17:2284. 10.3390/ijerph17072284 32231115 PMC7177676

[29] Zhang YF Zhang Y. Impact of environmental regulation on ecological efficiency under the background of new environmental protection law. J Environ Protect Ecol. (2020) 21:2296–304. 10.1016/j.ecolind.2021.108002

[30] Liu Y Liu M Wang GG Zhao LL An P. Effect of Environmental Regulation on High-quality Economic Development in China—An Empirical Analysis Based on Dynamic Spatial Durbin Model. Environ Sci Pollut Res. (2021) 28:1–18. 10.1007/s11356-021-13780-2 34018107

[31] Ma C He W. Local government competition and the environmental pollution. Financ Econ. (2016) 8:93–101.

[32] Wu H Hao Y Ren S. How do environmental regulation and environmental decentralization affect green total factor energy efficiency: evidence from China. Energy Econ. (2020) 91:104880. 10.1016/j.eneco.2020.104880

[33] Sigman H. Decentralization and environmental quality: an international analysis of water pollution levels and variation. Land Econ. (2014) 90:114–30. 10.3368/le.90.1.114

[34] Wu H Li Y Hao Y Ren S Zhang P. Environmental decentralization, local government competition, and regional green development: Evidence from China. Sci Total Environ. (2020) 708:135085. 10.1016/j.scitotenv.2019.135085 31812397

[35] Wallace EO. On the evolution of fiscal federalism: theory and institutions. Natl Tax J. (2008) 61:313–34. 10.17310/ntj.2008.2.08

[36] Goel RK Mazhar U Nelson MA Ram R. Different forms of decentralization and their impact on government performance: Micro-level evidence from 113 countries. Econ Model. (2017) 62:171–83. 10.1016/j.econmod.2016.12.010

[37] Sjoberg E Xu J. An empirical study of US environmental federalism: RCRA enforcement from 1998 to 2011. Ecol Econ. (2018) 147:253–63. 10.1016/j.ecolecon.2018.01.024

[38] Hao Y Xu L Guo YX Wu HT. The inducing factors of environmental emergencies: do environmental decentralization and regional corruption matter? J Environ Manag. (2021) 302:114098. 10.1016/j.jenvman.2021.114098 34794054

[39] Grooms KK. Enforcing the clean water act: the effect of state-level corruption on compliance. J Environ Econ Manag. (2015) 73:50–78. 10.1016/j.jeem.2015.06.005

[40] Konisky DM Woods ND. Environmental free riding in state water pollution enforcement. State Polit Policy Q. (2012) 12:227–51. 10.1177/1532440012438891

[41] Liu Y Hao Y Gao Y. The environmental consequences of domestic and foreign investment: evidence from China. Energy Policy. (2017) 108:271–80. 10.1016/j.enpol.2017.05.055

[42] Gray WB Shadbegian RJ. Plant vintage, technology, and environmental regulation. J Environ Econ Manag. (2003) 46:384–402. 10.1016/S0095-0696(03)00031-7

[43] Tang GP Li LH Wu DJ. Environmental Regulation, Industry Attributes and Corporate Environmental Investment. Acc Res. (2013) 83−9.+96. (in Chinese). 10.1036/j.cnki.ier.2013.06.005

[44] Wang R He X Diao X. Input-output efficiency of environmental protection enterprises and its influencing factors: an empirical analysis of 279 listed enterprises in China - ScienceDirect. J Clean Prod. (2021) 279:123652. 10.1016/j.jclepro.2020.123652

[45] Kou P Han Y. Vertical environmental protection pressure, fiscal pressure, and local environmental regulations: evidence from China's industrial sulfur dioxide treatment. Environ Sci Pollut Res. (2021) 28:1–16. 10.1007/s11356-021-14947-7 34152541

[46] Kesidou E Demirel P. On the drivers of eco-innovations: Empirical evidence from the UK. Res Policy. (2012) 41:862–70. 10.1016/j.respol.2012.01.005

[47] Arouri MH Caporale GM Rault C Sova R Sova A. Environmental Regulation and Competitiveness: Evidence from Romania. Ecol Econ. (2012) 81:130–9. 10.1016/j.ecolecon.2012.07.00131857422

[48] Dietz S Venmans F Cole MA. The endowment effect, discounting and the environment. J Environ Econ Manag. (2019) 97:67–91. 10.1016/j.jeem.2019.01.010

[49] Maxwell JW Decker CS. Voluntary Environmental Investment and R esponsive R egulation. Environ Res Econ. (2006) 33:425–39. 10.1007/s10640-005-4992-z

[50] Winner PH. Environmental regulation and investment: evidence from European industry data. Ecol Econ. (2011) 70:759–70. 10.1016/j.ecolecon.2010.11.01317302343

[51] Feng S Sui B Liu H. Environmental decentralization and innovation in China. Econ Model. (2020) 93:660–74. 10.1016/j.econmod.2020.02.048

[52] Chang HF Sigman H Traub LG. Endogenous decentralization in federal environmental policies. Int Rev Law Econ. (2014) 37:39–50. 10.1016/j.irle.2013.07.001

[53] Wang M. Environmental governance as a new runway of promotion tournaments: campaign-style governance and policy implementation in China's environmental laws. Environ Sci Pollut Res. (2021) 1–13. 10.1007/s11356-021-13100-8 33661500

[54] Liu YX Qi HJ Liu SQ. Margin Trading, Managers' Confidence and Corporate Environmental Investment. J Zhongnan Univ Econ Law. (2020) 8:1–12. (in Chinese). 10.19639/j.cnki.issn1003-5230.20200605.002

[55] Li H Zhao QW. Provincial environmental competition, internal control and corporate environmental protection investment: based on the study of two-stage intentional legalization. Financ Econ. (2020) 3:92–106. (in Chinese). 10.19692/j.cnki.issn1000-8306.20200301.015

[56] Zhang N Deng J Ahmad F Draz MU. Local government competition and regional green development in China: the mediating role of environmental regulation. Int J Environ Res Public Health. (2020) 17:3485. 10.3390/ijerph17103485 32429455 PMC7277232

[57] Treisman D. Defining and measuring decentralization: a global perspective Unpublished manuscript. Am Econ Rev. (2002) 5:38. 10.1017/j.jhazmat.2002.03.11

[58] Lutsey N Sperling D. America's bottom-up climate change mitigation policy. Energy Policy. (2008) 36:673–85. 10.1016/j.enpol.2007.10.018

[59] He Q. Fiscal decentralization and environmental pollution: evidence from Chinese panel data. China Econ Rev. (2015) 36:86–100. 10.1016/j.chieco.2015.08.01031745773

[60] Fredriksson PG Wollscheid JR. Environmental decentralization and political centralization. Ecol Econ. (2014) 107:402–10. 10.1016/j.ecolecon.2014.09.019

[61] Peng X. Is environmental decentralization conducive to industrial green transformation in China? Dynamic spatial effect test under the perspective of upgrading of industrial structure. Ind Econ Res. (2016) 2:21–31. (in Chinese). 10.13269/j.cnki.ier.2016.02.003

[62] Hosseini E Rezvani MH. E-customer loyalty in gamified trusted store platforms: a case study analysis in Iran. Bull Electr Eng Inf. (2021) 10:2899–909. 10.11591/eei.v10i5.3165

